# Therapeutic Potential of Targeting Mitochondria for Alzheimer’s Disease Treatment

**DOI:** 10.3390/jcm11226742

**Published:** 2022-11-14

**Authors:** Anna Atlante, Giuseppina Amadoro, Valentina Latina, Daniela Valenti

**Affiliations:** 1 Institute of Biomembranes, Bioenergetics and Molecular Biotechnologies (IBIOM)-CNR, Via G. Amendola122/O, 70126 Bari, Italy; 2 Institute of Translational Pharmacology (IFT)-CNR, Via Fosso del Cavaliere 100, 00133 Rome, Italy; 3 European Brain Research Institute (EBRI), Viale Regina Elena 295, 00161 Rome, Italy

**Keywords:** Alzheimer, neurodegeneration, mitochondrial dysfunction, mitochondrial bioenergetics, mitophagy, mitochondrial dynamics, mitochondrial biogenesis, therapeutic strategies

## Abstract

Alzheimer’s disease (AD), a chronic and progressive neurodegenerative disease, is characterized by memory and cognitive impairment and by the accumulation in the brain of abnormal proteins, more precisely beta-amyloid (β-amyloid or Aβ) and Tau proteins. Studies aimed at researching pharmacological treatments against AD have focused precisely on molecules capable, in one way or another, of preventing/eliminating the accumulations of the aforementioned proteins. Unfortunately, more than 100 years after the discovery of the disease, there is still no effective therapy in modifying the biology behind AD and nipping the disease in the bud. This state of affairs has made neuroscientists suspicious, so much so that for several years the idea has gained ground that AD is not a direct neuropathological consequence taking place downstream of the deposition of the two toxic proteins, but rather a multifactorial disease, including mitochondrial dysfunction as an early event in the pathogenesis of AD, occurring even before clinical symptoms. This is the reason why the search for pharmacological agents capable of normalizing the functioning of these subcellular organelles of vital importance for nerve cells is certainly to be considered a promising approach to the design of effective neuroprotective drugs aimed at preserving this organelle to arrest or delay the progression of the disease. Here, our intent is to provide an updated overview of the mitochondrial alterations related to this disorder and of the therapeutic strategies (both natural and synthetic) targeting mitochondrial dysfunction.

## 1. Introduction

After two decades and over 400 clinical trials focusing on the “one drug, one target, one disease” paradigm, there is still no effective therapy capable of blocking or at least slowing the progression of Alzheimer’s disease (AD), a highly prevalent neurodegenerative disorder with gradual cognitive decline and loss of memory, characterized by the accumulation of extracellular amyloid plaques (APs) and intracellular neurofibrillary tangles (NFTs) [1]. AD targets and destroys neuronal cells. Selectively, neurons in the hippocampus, an area of the brain associated with memory, appear to be particularly vulnerable. Currently, no neurodegenerative disease, not just AD, is curable and the available treatments only manage the symptoms or, at best, slow the progression of the disease. The fact that the pathophysiological process of AD actually begins many years before the clinical diagnosis of cognitive impairment provides an advantageous opportunity for potential interventions with preventive approaches and molecules capable of acting by modifying the course of the disease [2].

In the neurodegenerative progression of AD, long before neurons degenerate or die, APs, also called senile plaques, consisting mainly of a small peptide, β-amyloid (Aβ), begin to form in nerve cells. Aβ originates from the cleavage of the amyloid precursor protein (APP), a type I membrane glycoprotein of nerve cells, which plays a role, indeed multifunctional activities, so essential for neurons that transgenic mice with knockout for more than one member of APP family proteins (for APP and APLP1, APLP2) are lethal after birth [3]. The portion of APP that has been the subject of an increasing number of studies is the transmembrane part. APP is targeted first at the plasma membrane, then rapidly endocytosed to endosomes to be metabolized to Aβ or subsequently sent to the lysosomal compartment for degradation [4,5]. The fusion of endosomes with the cell membrane allows the secretion of Aβ_40_ and Aβ_42_ monomers into the extracellular matrix [6]. The ratio of the longer (Aβ42) to shorter (Aβ40) species is a critical factor determining amyloid fibril formation, neurotoxicity and progression of amyloid pathology in AD [7]. Although the majority of the peptides produced constitute the Aβ40 species, greater emphasis has been placed on Aβ42, which was detected at approximately 10-fold lower levels than Aβ40. A small elevation in the Aβ42/40 ratio increases the Aβ peptide’s aggregation properties, induces neurotoxicity and alters synaptic activity in primary neurons [8]. On the other hand, it was suggested that Aβ40 might serve as a protective factor in AD progression, mediating the inhibition of Aβ42 fibrillogenesis [9]. Collectively, these data suggest that an elevated Aβ42/40 ratio is linked to AD pathogenesis and could be targeted therapeutically. 

More or less at the same time as the anomalous demolition of APP begins, within the same neurons, the Tau protein undergoes a series of post-translation modifications and attacks by proteolytic enzymes that end with its partial degradation and constitution of spiral products, easily identifiable under an electron microscope, the NFTs [10]. Researchers attribute Aβ accumulation as a marker for early-stage disease and phosphorylated Tau (p-Tau) as a marker for late-stage disease [11]. Their research findings also showed that p-Tau increases with the Aβ fraction and speculate that Aβ may be involved in Tau phosphorylation [11]. Concerning this, several hypotheses related to the pathogenesis of AD were studied and the Aβ cascade and the hyperphosphorylation of the Tau protein are the two main hypotheses [12]. The Aβ cascade hypothesis states that Aβ, deposited in the form of neuroinflammatory plaques, induces Tau pathology and facilitates the development of AD by damaging neuronal cells [13]. These theories on the development and presence of extracellular APs and Tau protein agglomerates in brain tissue have always assumed an important and prestigious role, leaving the others “in the shadow”, in the attention of global research in this field. Experimental pre-clinical and clinical studies carried out the last 25 years proposed the “Amyloid Cascade” as the leading hypothesis of AD pathogenesis. It posits that the increase in amyloid-β (Aβ) is the key event in AD that triggers downstream the Tau pathology (hyperphosphorylation, truncation and misfolding) leading to early synaptic deterioration and, then, memory impairment followed by late neuronal death. According to this point of view, Aβ is the *primum movens* in a sequential cascade of pathological events that places downstream Tau as the crucial mediator of neuronal demise [14]. More recently, an alternative and not mutually exclusive “dual-hit hypothesis” has been proposed by which Aβ and Tau are partners in crime by cooperating in damaging the synapses, thus resulting in memory loss and “dying back” neuronal death [15].

In particular, from the mid-1980s to today, the volume of research aimed at defining the role of Aβ in AD has been enormous, just as the discussion on the other aspect of fibrillar disease is equally likely. At present, the research aims to understand the reasons why in the brain of the patient the two proteins, normally present in the brain of a healthy subject, undergo erroneous synthesis or abnormal metabolisms until they accumulate. Among other things, if until a few years ago it was APs that were considered harmful; now, a plethora of studies indicate that these clusters can also be present in people in perfect health [16]. In fact, according to more recent studies, the soluble species that disintegrate from the plaques are the dangerous ones, while the formation of the plaque could even represent a defense mechanism as long as it holds up [17,18,19]. The topic is, therefore, very complex, but extremely relevant if we only think that all these discoveries that add new pieces to the intricate puzzle of events and aim at understanding the causal links and timeline leading to the disease are the pillars on which the therapies of the future will rest. In this regard, as already mentioned above, in the current state of knowledge, there is no cure for AD; that is, we do not have a causal treatment that consists in the removal of the cause of the disease, but only “symptomatic” drugs, i.e., aimed at attenuating the clinical manifestations of the disease. Only six drugs are approved by the United States Food and Drug Administration (FDA) for the treatment of symptoms of AD: four acetylcholinesterase inhibitors (tacrine, donepezil, galantamine and rivastigmine), an N-methyl-D-aspartate receptor antagonist (NMDA) (memantine) and a fixed combination of donepezil and memantine [20,21,22]. However, none of these drugs are effective in modifying the biology behind AD and nipping the disease in the bud. The theoretical assumption for the use of acetylcholinesterase inhibitors lies in the finding of a cerebral deficiency in AD of the chemical substance acetylcholine—a neurotransmitter important for memory and thinking—which sends messages from one cell to another and, after having finished its task, it is degraded by the acetylcholinesterase enzyme so that it does not accumulate between cells. Acetylcholinesterase inhibitors prevent the destruction of acetylcholine and compensate—without stopping—the destruction of cells caused by the disease, as well as improve some cognitive symptoms (such as memory and attention) and behavioral symptoms (such as apathy, agitation and hallucinations), but this capacity gradually decreases with the progression of the disease [23,24,25,26]. The other type of drugs, those used in the 1990s, are based on the use of agonists of muscarinic or nicotinic receptors [27,28]. In this regard, epidemiological studies have shown a delay in the frequency of AD in smokers, in which the inhalation of nicotine would exert an action comparable to that obtained during therapeutic treatment based on agonists of the same nicotinic cholinergic receptors [29].

In any event, if the goal is to remove the cause, the most obvious way to stop dementia is certainly to limit the accumulation of Aβ in the tissue by (i) stimulating its disposal and/or (ii) decreasing its production. In the first case, the purpose was achieved using this peptide, i.e., Aβ, to vaccinate transgenic mice capable of producing large amounts of anti-Aβ [30,31] antibodies. Compared to the controls, the vaccinated animals were able to perform more difficult exercises, suggesting that, having less amyloid in the brain, the neuronal degeneration was also less. On the other hand, in order to avoid the overproduction and accumulation of Aβ, the activity of β- and γ-secretases could, in theory, be reduced, but in practice, the development of anti-β-secretase substances is made problematic by the possibility that inhibitors act simultaneously on multiple substrates. For example, heparin is an in vitro inhibitor of β-secretase [32], but, having been used for some time as an anticoagulant, it is unwieldy for long-term treatment. Under experimental conditions, the production of Aβ42 was also limited by some non-steroidal anti-inflammatory drugs (NSAIDs), such as ibuprofen, indomethacin and sulindac, drugs that favor its transformation into a shorter and non-neurotoxic peptide. However, the use of anti-inflammatories was found to be ineffective in containing cognitive impairment [33].

After 20 years of studies, struggles and failures, the change of course towards the use of monoclonal antibodies (mAbs) seems to be an option that gives hope at the moment. It is a therapeutic approach aimed at the development of precision drugs capable of altering the mechanisms underlying the disease; that is, for example, reducing the levels of Aβ in patients with AD by (i) inhibiting the generation of Aβ [34], (ii) reducing the levels of soluble Aβ (for refs, see [35]), (iii) improving the clearance of Aβ from the brain (for refs, see [35]). Currently, several laboratories are actively involved in immunotherapy research to eliminate soluble and insoluble Aβ from the brain; consistently, Aβ immunization in transgenic AD mouse models demonstrated that Aβ levels can be reduced in the brains of AD mice (for refs, see [35]). Aducanumab, a mAb with selective action on aggregated forms of Aβ, is the first to be approved by the FDA for the treatment of AD [36]: it has the ability to reduce the Aβ overload of the brain and, in theory, to limit, block or prevent the damage caused by Aβ, while reducing p-Tau. Importantly, it intervenes not so much on the symptoms, but on the pathophysiological processes that seem to be at the origin of the development of the disease [36,37]. However, at present, neuroscientists are still skeptical because there is no absolute certainty that the reduction in Aβ plaques can be correlated with actual patient improvement [38,39]. Furthermore, based on in vitro and in vivo evidence supporting the crucial role of Tau modifications in driving AD progression [40], administration of another mAb, called 12A12, turn out to be greatly effective in different preclinical AD animal models leading to ‘significant improvements’ of the main alterations associated with the disease. 12A12 has a very high specificity for a toxic AD-relevant Tau fragment that is capable of causing alterations mainly at the mitochondrial level [41]. This antibody has been developed by the research group coordinated by Pietro Calissano, longtime collaborator of the Nobel laureate Rita Levi-Montalcini, at the Ebri Foundation. The data obtained are very encouraging: a significant recovery of cognitive memory deficits, a reduction in Aβ level and a restoration of molecular mechanisms related to the action of insulin in the brain, which then favored reparative processes both of mitochondria and oxidative stress (OS), are detected by administering the 12A12mAb, which neutralizes the pathogenic Tau form, for two weeks in three different preclinical, both genetic and sporadic AD animal models, such as Tg2576 expressing the human double Swedish mutation (K670M/N671L) of APP695 isoform, 3xTg-AD carrying the APP Swedish, MAPT P301L and PSEN1 M146V transgenes and Streptozotocin(STZ)-induced diabetic mice [41,42].

The idea of exploiting the mechanisms of the immune system to prevent the formation of Aβ-positive plaques and Tau agglomerates or otherwise to stimulate their elimination is an approach in which great hopes are placed for the treatment of AD. However, one cannot overlook a problem that has long plagued the research in the field, namely the inconsistency of the “amyloid cascade” theory, according to which elderly patients, with heavy loads of APs in the brain, lack measurable cognitive impairment, while other patients who show little or no amyloid accumulation manifest severe AD dementia [16,43]. In addition to this fact, there is the evidence that a series of anti-amyloid drugs do not provide any benefit to patients in clinical trials or halt the inexorable cognitive decline caused by the disease. Furthermore, it is increasingly evident that plaques and tangles are lagging behind in the devastating sequence of events that culminates in AD [44].

As things stand, researchers have turned to look for other processes that occur in the very early stages and that can trigger the disease. One of the most promising pathways of the new research focused on the role of mitochondria dysfunctional activity, which places these power plants inside the cell at the center of the destructive action taking place in AD neurons [45,46].

In AD, the mitochondrion does not function as it should, thus compromising some fundamental processes of the nerve cell. Importantly, mitochondrial dysfunctions not only occur in the initial stages of AD, but even before clinical symptoms. Various experimental models of the disease show an altered functioning of mitochondria which, therefore, play a crucial role in triggering degenerative processes, causing extensive oxidative damage of neuronal lipids, of nucleic acids and proteins (for refs, see [35]) in the brains of AD patients. Growing evidence suggests that in AD free radicals, generated by mitochondria and transported to the cytoplasm, activate secretase and facilitate the cleavage of the APP molecule along the amyloidogenic route (for refs, see [35]). The β- and γ-cleaved APP molecule, i.e., Aβ, generates other free radicals, leading to the disruption of electron transport chain (ETC) and enzymatic activities, inhibition of ATPase and subsequent oxidation of both nuclear and mitochondrial proteins. Damage generated by mitochondria causes neurodegeneration and cognitive decline in patients suffering from AD [45,46,47,48]. OS markers, such as 8-hydroxyguanosine and hemeoxygenase, have been located in pathological lesions in the brains of AD-affected subjects [49,50].

Impaired mitochondrial function would not only precede the accumulation of Aβ and Tau in mouse models of the disease but may also activate processes that cause the dangerous stores of these two toxic proteins, endorsing the idea that the destruction of defective mitochondria could be a pivotal way to cure the disease. However, the latter hypothesis could be objected because cells normally get rid of defective mitochondria through mitophagy, a process that selectively sequesters damaged or depolarized mitochondria into double-membraned autophagosomes for subsequent lysosomal degradation [51]. Additionally, adding insult to injury, mitophagy appears to be weakened in AD brains. Improving, i.e., by ‘adjusting’, this mechanism, the symptoms of dementia also seem to be drastically attenuated, at least in animal models [52,53]. Another question to consider is that, whether or not mitochondrial dysfunction is a hallmark of AD, the connection of the two AD-associated proteins, i.e., Tau and Aβ, to mitochondria is equally certain and extremely interesting. Here, we report only some of the experimental data that have ascertained this existing relationship, i.e., between mitochondria and toxic Aβ and Tau.

As early as 20 years ago, Amadoro et al. [54] reported that an intracellular 20–22 kDa NH_2_ truncated Tau fragment was largely enriched in the mitochondria of human AD brain synaptosomes and that the amount of Tau in the terminal fields correlated with both pathological synaptic changes and impairment functional organelles. In particular, the pathophysiological linking between the overexpressed N-terminal Tau fragments (1–25 aa and 26–44 aa) and mitochondrial function has been investigated by Atlante et al. [55]: mitochondrial oxidative phosphorylation (OXPHOS) machinery is not affected by the N-terminal fragment 1–25 aa Tau fragment, but is dramatically altered by the N-terminal fragment 26–44 aa Tau, with adenine nucleotide translocator (ANT) as the sole mitochondrial target [54,55]. In line with this, studies conducted by David et al. [56] have shown that p-Tau can promote mitochondrial dysfunction in neurons of FTLD Tau (P301L) mutant transgenic (pR5) mice by altering ETC complex I. Aged P301L Tau mice with significant accumulation of p-Tau show reduced mitochondrial respiration, reduced V-complex activity and higher reactive oxygen species (ROS) levels than wild-type controls [56].

As for Aβ, the observation that cells lacking mitochondrial DNA, i.e., devoid of critical subunits of the respiratory complexes, are not sensitive to the toxic effects of Aβ primarily suggest that the toxicity of Aβ makes use of functional ETC [57]. It is not surprising that, even for this extracellular localization protein, the most frequent targets (site(s) of action) are precisely the mitochondria (for refs, see [58]). During the processing of APP, it happens that any Aβs which are not degraded can remain inside the cytoplasm, or—even—dissociate from the formed extracellular plaques and re-enter the cells where they can be deleterious. Lustbader et al. [59] demonstrated that Aβ is localized in the mitochondria in the neurons of AD transgenic mice. Consistently, Manczak et al. [60] have proved that Aβ accumulates within the mitochondria of AD patients and transgenic mice, causing both a selective defect in Complex I activity, an increase in ROS and an impairment of Complex IV. Specifically, Bobba et al. [61] have shed light on the molecular mechanism responsible for the alteration of ETC induced by Aβ: the defect in the activity of Complex I, caused by Aβ1–42, prompts an increase in ROS which is, in turn, responsible for the impairment of Complex IV (COX), possibly due to lipid peroxidation of the membranes. Consistently, Crouch et al. [62] have found that synthetic Aβ42 inhibits the COX terminal complex in a way that depends on the presence of Cu^2+^, suggesting that endogenous Aβ is associated with brain mitochondria and that synthetic Aβ (1–42) is a potent COX inhibitor. The Aβ peptides themselves interact with an NH_2_-derived neurotoxic Tau fragment of the human Tau40 isoform (441 amino acids) in human AD synapses in association with mitochondrial ANT, a condition that further worsens mitochondrial dysfunction by aggravating the impairment of the ANT, thus leading to a strong energy metabolism deficit [46,54,55]. Aβ peptides have also been shown to interact with mitochondrial matrix components altering mitochondrial dynamics by differentially modulating fission/fusion proteins (for refs, see [63]). Finally, the effects of Aβ aggregation can be mitigated by efficient mitochondrial proteostasis (for refs, see [63]).

Regarding Aβ and Tau localization, it has been largely demonstrated that both proteins can influence the mitochondrial function at different levels (transport, morphology and mitochondrial bioenergetics) by acting either inside or outside the mitochondria themselves [64,65]. This important finding adds an additional layer of complexity of the interaction of the toxic Alzheimer’s proteins on mitochondria.

In addition to the detrimental effect of Aβ and Tau on mitochondrial function, APP-derived C-terminal fragments (APP-CTFs) also contribute to the progression of AD by acting on mitochondria. Concerning this, Lee et al. [66], using induced neural stem cells (iNSCs) from AD patient-derived fibroblasts, demonstrated that APP-CTFs accumulation resulted in mitophagy failure, as validated by increased LC3-II and p62 and inconsistent PTEN-induced kinase 1 (PINK1)/E3 ubiquitin ligase (Parkin) recruitment to mitochondria and failed fusion of mitochondria and lysosomes. Consistently, Vaillant-Beuchot et al. [67] demonstrated that APP-CTFs accumulation either triggered by pharmacological blockade of its cleaving enzyme γ-secretase or after its overexpression leads to mitochondrial size alteration and cristae disorganization associated with enhanced mitochondrial ROS production, and mitophagy failure phenotype in cells. Similarly, these alterations were observed in γ-secretase inhibitor-treated young 3xTgAD mice. Further, the accumulation of an APP-CTF, called C99, a direct precursor of Aβ, in areas vulnerable to AD neurodegeneration neurons [68] results in an altered lipid composition of mitochondrial membranes which interferes with the proper assembly and activity of mitochondrial respiratory supercomplexes, thereby likely contributing to the bioenergetic defects characteristic of AD [69]. Ultimately, being both the presenilic γ- secretase activity and its direct substrate (i.e., C99) located in mitochondria in their endoplasmic reticulum (ER) membrane (MAM) domains [69,70], it can be inferred that the former can cleave the latter, thus mediating the generation of Aβ.

After working for approximately thirty years on the role of mitochondrial bioenergetics in the mechanisms responsible for the onset and progression of AD [46,55,62,71,72,73,74], here and now, our intent is to collect data from studies carried out by our colleagues—who have also poured out their commitment and expertise—in order to provide an overview of mitochondrial alterations linked with this disorder and therapeutic strategies (both natural and synthetic) targeting mitochondrial dysfunction and OS and capable of preferentially accumulating in the brains of affected patients. Understanding the mechanisms underlying these dysfunctions is fundamental and opens up captivating scenarios for the development of therapies and/or approaches aimed at preserving this organelle to arrest or delay the progression of the disease [75]. However, since mitochondrial functions are closely related to each other, the alteration of each of them could develop neurodegeneration, making it difficult to identify what is the critical change.

## 2. The Mitochondrion: Its Dysfunctions in AD

Abnormal mitochondrial structure, accumulation of damaged mitochondria, impaired bioenergetics and metabolic changes, along with excessive ROS production, are well documented in various AD models [76], thus providing potential molecular targets for the treatment of this neurodegenerative illness.

**Figure 1:**
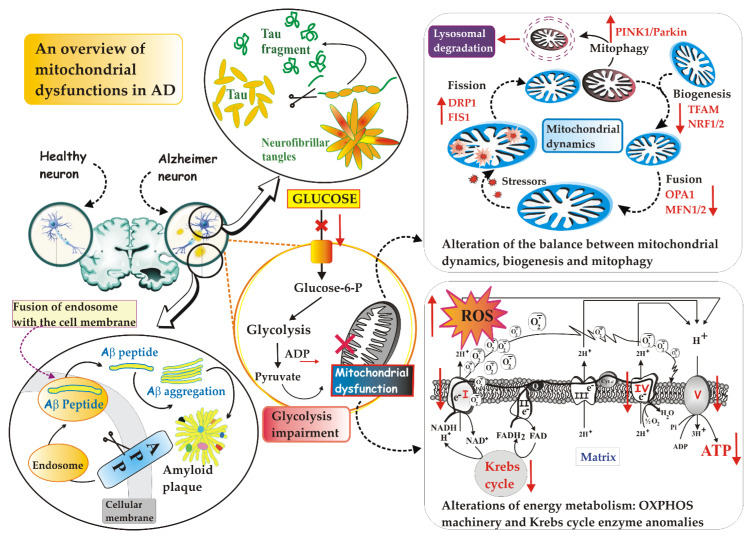
An overview of mitochondrial dysfunctions in AD. Although there are different localizations of toxic proteins, i.e., intra- vs. extracellular protein for Tau and Aβ, respectively, both can influence the mitochondrial function at different levels (transport, morphology and mitochondrial bioenergetics). For details, see the text. Abbreviations: Aβ, β-amyloid; APP, amyloid precursor protein; DRP1, dynamin-related protein 1; FIS1, mitochondrial adaptor fission 1; MNF1/2; mitofusin 1 and 2; NRF1/2, nuclear respiratory factors 1 and 2; OPA1, optic atrophy 1; PINK1, PTEN-induced kinase 1; ROS, reactive oxygen species; TFAM, mitochondrial transcription factor A.

The maintenance of a functional mitochondrial population during the life of neurons is of paramount importance and involves a fine regulation of mitochondrial dynamics, maintained by the coordinated interplay between mechanisms of mitochondrial fusion and fission [77,78], biogenesis [79] and mitophagy [80] (Figure 1). Thus, it is easy to imagine how the interruption of any of these mitochondrial processes has a strong impact on cellular viability and functions, since neurons in particular, on the one hand, require an incredibly high amount of energy for neurotransmission, and on the other, they need an efficient system to eliminate damaged mitochondria (for refs, see [63]) and reduce ROS-induced death [63,81].

Many studies have been carried out to identify dysfunctions affecting the quality control mechanisms of mitochondria (for refs, see [82]), giving clear evidence that all involved steps are impaired in AD pathogenesis (for refs, see [83]). 

### 2.1. Defective Mitochondrial Dynamics in AD

It has been known for decades that mitochondria continually undergo fusion and fission events. These two processes, which collectively go by the name of “mitochondrial dynamics”, are in constant evolution and depend on the state and needs of the cell [84]. Highlighted by a fluorescent dye, mitochondria are neon dashes that move slowly in the dense internal broth of cells: they stretch and contract, merge with each other and divide again, stand side by side and then fall apart and they separate, with the clear desire to enter into relationship with each other. In the specific case of neurons with extended axons and numerous dendrites, repositioning the organelles where more energy is required [85] is a vital feature. These mitochondrial encounters serve several purposes, of which at least one appears to be collaborative. Mitochondria that have no genome or whose genetic material is dysfunctional can be “saved” by fusion with healthy organelles [86]. The interconnections create a channel through which chemical signals and proteins can be shared; it is also possible that fusion helps to distribute proteins more evenly among the various mitochondria.

Optic atrophy 1 (OPA1)—a mitochondrial GTPase—and mitofusin 1 and 2 (MFN1 and MFN2), the three main fusion proteins, modulate the fusion of the outer and inner membrane of adjacent mitochondria [84,87]: a decrease in their activity has been observed in the post mortem tissue of AD patients [88]. On the contrary, dynamin-related protein 1 (Drp1), also belonging to the GTPase family, plays a key role in mitochondrial fission, a process which, by generating smaller mitochondria with different properties, separates the damaged from the functional mitochondria. Drp1 is a cytosolic protein that translocates on the mitochondrial outer membrane (MOM) of damaged and dysfunctional mitochondria and induces their division [84,89]. Drp1, together with mitochondrial adaptor fission 1 (Fis1), a protein indirectly involved in mitochondrial fission via binding Drp1, forms a ring structure that constricts the mitochondrion until it splits in two [90]. Abnormal expression of Drp1 has been observed in post mortem AD brains, as well as in AD mouse models and APP-overexpressing cell lines [91]. P-Tau and Aβ increase the phosphorylation of Drp1 and the translocation of Drp1 into the mitochondria in order to increase fission, with an inevitable fatal outcome: fragmented mitochondria increase and mitophagy is then activated (for refs, see [92]). Even mild OS instigates increased Drp1-dependent fission that promotes mitophagy [92].

In summary, on one hand, the mitochondrial fusion allows two adjacent and smaller mitochondria to merge and form larger and more elongated mitochondria that are, therefore, more productive in terms of cellular energy generation, capable of exchanging mitochondrial DNA (mtDNA) and of diluting the possible damage of the organelle [93]. On the other hand, mitochondrial fission causes the fragmentation of the mitochondrial network, causing an increase in ROS production and signaling of cell death, but also allows a correct mitochondrial division and the isolation of damaged organelles, which will then be degraded by mitophagy [94].

In post mortem AD brains, cellular and mouse models of APP, by using co-immunoprecipitation and colocalization studies, researchers found that Aβ, interacting with Drp1, causes increased mitochondrial fragmentation and reduced fusion and, consequently, a decrease in OXPHOS [95]. The increased expression of proteins related to mitochondrial fission is also related to the accumulation of Tau proteins in neurons derived from AD patient-derived, pluripotent stem cells (iPSC) [87].

In other studies (for refs, see [96]), the interaction of p-Tau with Drp1 in post mortem AD brains and in mouse models of Tau in disease progression has been deeply investigated. Using cortical protein lysates from severe AD brain tissue, pathogenic forms of Tau have been found to cause dysregulation of Drp1 function—precisely, p-Tau co-precipitates with Drp1 [97]—resulting in abnormal mitochondrial dynamics [98,99]. This study has been further validated using brain tissue from APP/presenilin1 (PS1) mice, 3xTg-AD mice (aged 13 months) and age-matched wild type controls [97]. These effects are associated with significantly increased levels of GTPase activity in cerebral cortex tissues from APP/PS1 and 3xTg-AD mice, which is likely to promote the increased mitochondrial fragmentation detected in AD neurons [97]. Imbalance of mitochondrial fusion and fission proteins in AD is, therefore, proposed as an early event of disease progression [100,101] and, in this framework, several pieces of evidence show changes in fusion and fission protein levels towards augmented mitochondrial fission in patients’ brain tissue with AD [100,101], an event that leads to the degradation of the same organelles by mitophagy [102]. The increase in mitophagy has been observed in SH-SY5Y cells after incubation with Aβ, which itself may be a trigger for mitophagy (for refs, see [102]). This finding has been suggested by the fact that in primary mouse neuronal cultures, exposure to oligomeric Aβ42 induces transcription imbalances of genes encoding mitochondrial fission and fusion proteins, leading to increased mitochondrial fission and autophagy [102]. Overall, too much fission leads to ROS over-production, mitochondrial dysfunction and defective mitophagy in AD (see [96]).

Taken together, these studies strongly indicated that inhibition of fission-related proteins or overexpression of fusion-related ones may represent possible therapeutic treatments for AD neurodegeneration [103]. 

### 2.2. Defective Mitophagy in AD

There are two main types of mitophagy, i.e., PINK1/Parkin-dependent and independent [104], which require the involvement of different proteins.

The PINK1/Parkin route is one of the most characteristic routes. In healthy mitochondria, PINK1, which contains a mitochondrial targeting sequence (MTS), is recruited by the mitochondrion, crosses the outer and inner membranes via the TOM and TIM complexes, becoming a transmembrane protein at the level of the mitochondrial internal membrane (MIM), and is degraded by proteases present in the mitochondrion. These processes keep the PINK1 concentration below a certain threshold value in healthy mitochondria. In contrast, damaged mitochondria lack the membrane potential needed for import, so PINK1 accumulates on the MOM, marking the damaged mitochondrion for removal. PINK1 recruits the E3 Parkin ubiquitin ligase [105], activating it, thus allowing Parkin-mediated ubiquitination of other proteins. Some of these proteins include Mfn1/Mfn2 and mitoNEET. The ubiquitination of proteins present on the surface of the mitochondria acts as an initiating factor for mitophagy. When the increase in the ubiquitin signal on the surface of the mitochondria occurs, a membrane called the phagophore is formed near or even directly around depolarized mitochondria. The phagophore expands until its edges fuse around its target(s), forming a double-membrane structure called an autophagosome. Next, the autophagosome fuses with a lysosome and the contents are degraded by lysosomal enzymes [106].

Changes in mitophagy have been reported to lead to a gradual accumulation of defective mitochondria in AD [107]. In detail, an unbalanced increase in ROS levels occurs under AD conditions, together with a lowering of antioxidant mechanisms, which eventually causes OS. This evidence suggests that the aberrant accumulation of dysfunctional mitochondria in AD neurons is probably attributable not only to an inadequate mitophagic capacity, but also to the inability to counteract OS. In support of this finding, cytoplasmic accumulation of mtDNA and organelle proteins is a hallmark of AD neurons showing a rise in oxidative damage [107]. In addition, an increase in PINK1 and Parkin in the early and later stages of the disease, respectively, has been reported in the AD hippocampus, together with an upregulation in markers of mitochondrial content [107].

Taken together, these and other in vitro and in vivo studies suggest that altered mitophagy (for refs, see [108])—baseline mitophagy levels reduced by 30–50% in post mortem hippocampal brain samples of AD patients, compared to normal [109]—could be both an upstream and downstream step in the nefarious appearance of Aβ and Tau along a vicious circle that causes synaptic dysfunction and cognitive deficit [107]. Consistently, retrograde transport of damaged distal mitochondria to the neuronal soma, where they should be degraded in mitolysosomes, is also impaired in AD patients [107].

Mitophagy was also found impaired in the peripheral fluids of AD patients, as evidenced by lowering serum protein levels of Parkin (see [107]), associated with decreasing Parkin and increasing PINK1 and LC3 mRNA levels in peripheral blood (see [107]). Consistently, Parkin overexpression attenuates Aβ-induced fragmentation and mitochondrial dysfunction, as demonstrated by increased membrane potential (Δψm), ETC complex activity and ATP level, as well as decreased ROS production [110], thereby alleviating the toxic insults due to mitochondrial impairment associated with AD. Regarding this, of interest is the study conducted by Fang et al. [111] in both Aβ and Tau *Caenorhabditis elegans* models of AD. The insertion of genes that trigger the expression of Aβ and Tau in the worm—a procedure capable of mimicking key AD features—and the subsequent activation of mitophagy effectively removed the damaged mitochondria, reduced the levels of Aβ and p-Tau and improved memory. Fortunately, the scientists have found that, in addition to worms, mice treated with the mitophagy activator are able to better remember the context associated with a stimulus in several learning tests, thus suggesting that efficient regulation of mitochondrial dynamics alleviates symptoms related to the disease, improves learning and reduces memory deterioration.

On the contrary, the excessive activation of mitophagy would eliminate too many mitochondria—perhaps even the ‘healthy’ ones—causing energy shortages and other deleterious effects. However, while this study has exciting potential, mitophagy activation globally could have undesirable targets on healthy and functional mitochondria, especially in other areas that are not in the brain. From these studies emerges the idea of using the molecular protagonists of mitophagy as peripheral biomarkers of AD.

### 2.3. Defective Mitochondrial Biogenesis in AD

Opposite to mitophagy is mitochondrial biogenesis [112,113]. They are complementary events, sharing signaling pathways and working in a coordinated manner to remove damaged mitochondrial material and add new functional mitochondrial mass. By creating a cycle of degradation and reconstitution of new mitochondria, the two processes work to preserve the optimal functionality of the intracellular mitochondrial pool. The term mitochondrial biogenesis refers to the process of generating new mitochondria. It is a complex and regulated genetic process requiring the use of nuclear DNA to encode mitochondrial proteins. Without a doubt, the best-known regulators of mitochondrial biogenesis are the peroxisome proliferator-activated receptor gamma 1-alpha coactivator (PGC-1α) transcription coactivator, the nuclear respiratory factors NRF-1 and NRF-2 and mitochondrial transcription factor A (TFAM) [92], i.e., proteins with the ability to bind to DNA in a certain area to regulate transcription, both through its activation and its inhibition.

Briefly, biogenesis is induced by PGC-1α which activates NRF-1 and NRF-2—they upregulate nuclear genes that encode subunits of mitochondrial complexes I–V [114]—that in turn, activate TFAM (for refs, see [95]). TFAM translocates into mitochondria and binds to mtDNA to regulate replication, transcription and stability [115]. Accumulated evidence suggests the regulatory role of sirtuin 1 (SIRT1) and AMP-activated protein kinase (AMPK) in the mitochondrial biogenesis upstream of PGC-1α. SIRT1, a NAD^+^ -dependent deacetylase protein, can directly deacetylate PGC-1α and increase its transcriptional activity [116]. AMPK, activated by increasing the AMP/ATP ratio, phosphorylates PGC-1α to promote mitochondrial biogenesis in addition to its effect in promoting glucose transport and fatty acid oxidation.

Mitochondrial biogenesis in AD neurons is impaired [117]: the number of mitochondria and the levels of NRF-1, NRF-2 and TFAM together with the nuclear levels of PGC-1α are reduced in the AD hippocampal tissues and also in M17 cells expressing the human double Swedish mutation of APP695 isoform (APPswe M17 cells) (for refs, see [92]). Thus, altered mitochondrial biogenesis can induce mitochondrial dysfunction in AD [117].

### 2.4. Defective Mitochondrial Energy Metabolism in AD

Patients with AD exhibit early metabolic deficits associated with impaired mitochondrial function and aberrant accumulation of damaged mitochondria (for refs, see [118]), prior to the emergence of any histopathological or clinical abnormalities (for refs, see [118]).

It is certain that energy metabolism is impaired in AD [63,119]. Consistently, the activities of mitochondrial respiratory complexes [79,120], as well as pyruvate dehydrogenase complex, isocitrate dehydrogenase, α-ketoglutarate dehydrogenase and ATP synthase activities were found to be reduced [63]: this state of affairs is associated with mitochondrial impairment of Δψm and OXPHOS [121], reduced intracellular ATP levels and increased OS [122], which intensifies deposition of amyloid aggravating cognitive dysfunction in AD mice and contributes to the phosphorylation and polymerization of Tau [123].

Furthermore, studies performed using fludeoxyglucose–positron emission tomography (PET) show that glucose hypometabolism—caused by reduced glycolysis, synaptic dysfunction and neuronal loss [124]—is markedly evident in the hippocampus and cortical regions [125], predates the clinical diagnosis of AD by anticipating the onset of histopathological features and symptoms by decades [63,126] and predicts cognitive decline in normal aging, as well as the progressive cognitive decline from mild cognitive impairment to AD stage (for refs, see [63]).

In this regard, Bobba et al. [127] investigated how, in the various phases of AD disease, glucose metabolism varies. They observed that in the early phase of apoptosis, which mimics the development of AD together to the late phase, glucose metabolism is enhanced, i.e., key proteins internalizing and metabolizing glucose, such as glucose transporter, hexokinase and phosphofructokinase are up-regulated, in concomitance with a parallel decrease in oxygen consumption by mitochondria and increase in L-lactate accumulation. The aerobic glycolysis, i.e., Warburg effect, is essentially due to the protective numbness of mitochondria. Interestingly, these nerve cells, exhibiting a shift in glucose metabolism to favor lactate production, are designated for resistance against Aβ toxicity [128] and, as a result, mitochondrial-derived ROS, closely related to Aβ toxicity, diminished in resistant-cells with respect to sensitive ones [74]. Understanding how nerve cells become resistant to Aβ toxicity will be central to understanding how some nerve cells within the AD brain are able to survive while the majority die. Although the physiological relevance of these findings in an in vivo AD model is unknown, the characterization of the mechanisms by which glycolysis is upregulated in Aβ-resistant cells could reveal possible targets for drug therapy in the treatment of AD in the early stages. A salient fact is that aerobic glycolysis—measured in humans by PET scan—is a good predictor of damaged regions in AD and of disease development [129,130].

In the late phase of apoptosis, the loss of the adaptive advantage afforded by aerobic glycolysis exacerbates the pathological processes underlying neurodegeneration, inevitably leading the cell to death. Fluorodeoxyglucose–PET studies have shown that the decline in brain glucose metabolic rate occurs in the prodromal stages of AD and is further exaggerated after disease onset [131].

Cerebral glucose hypometabolism and insufficient mitochondrial energy generation contribute to synapse loss and impair clearance of the Aβ42 and Tau proteins from the brain (for refs, see [132]). Conversely, the accumulation of Aβ42 and Tau triggers mitochondrial damage, alters energy production and increases OS [132]. Furthermore, neurotoxic proteins also inhibit glucose transporter type 4 (GLUT4) [133] and phosphofructokinase, thus blocking glucose uptake, aerobic glycolysis and ATP synthesis. These data give just an idea of the teamwork that causes neurodegeneration, supporting the hypothesis that mitochondrial dysfunction is the initial trigger for AD (for refs, see [82]) making it urgent to develop effective drugs that alleviate the bioenergetic deficits in vulnerable neurons from the most AD-affected brain regions.

## 3. Therapeutic Strategies Targeting Mitochondria for the Treatment of AD

Strategies focused on reducing toxic proteins, i.e., Aβe Tau, have greatly failed in clinical trials. New targets and therapeutic strategies are urgently needed. Mitochondria are considered a potential therapeutic target for AD [134,135].

Mitochondrial function already plays a significant role in AD in the early stage of the disease manifestation. Hence, the regulation of mitochondrial deficits has emerged as an attractive and useful strategy for inhibiting and/or slowing the neurodegenerative process in AD and other brain disorders. Overall, current preventive and/or therapeutic strategies for targeting mitochondrial dysfunction primarily focus on antioxidants, anti-apoptotic agents and agents that improve glucose metabolism and mitochondrial bioenergetics [136,137,138]. Many of these approaches have shown clear cognitive benefits in preclinical investigations in AD models [138,139,140]. However, in the clinical practice, most interventions have not been adequate in preventing, delaying or even reversing the cognitive decline [141,142]. Probably this is due to (i) the lack of valuable in vitro and in vivo animal models able to fully recapitulate the pathophysiological conditions of AD; (ii) the objective limitations of the potential therapeutic molecules, such as their poor bioavailability, poor penetration through the blood–brain barrier (BBB) and a limited sustenance of the half-life of dose-response activities. Therefore, more recent attempts have been made to overcome these drawbacks by anchoring the candidate molecule and/or its derivative to lipids, micelles, nanoparticles, liposomes, metal complexes and other entities, as we will see below.

To be totally realistic, targeting mitochondria can have consequences, not only unwanted, but also missed outcomes. A therapy aimed at mitochondria will have to take into account individual risks, in particular genetic or non-genetic factors that affect energy metabolism as well as the different bioenergetic phenotypes between different types of brain cells and their dynamic adaptation through the stages of the disease. The use of molecules endowed with antioxidant action, in combination with current pharmacological treatments, will aim to improve mitochondrial metabolism and, therefore, the production of ATP through the pharmacological stimulation of mitochondrial biogenesis, mitophagy, dynamics and concomitant detoxification of ROS. This is not a strategy that solves the root problem, but it certainly could improve the daily life quality of patients and, then, of their caregivers.

Below, we propose therapeutic strategies targeting mitochondria, attempting to distinguish between the different types of mitochondrial injury that occurs in AD.

### 3.1. Therapeutic Strategies Targeting Mitochondrial Bioenergetics 

The decrease in brain bioenergetics induced by mitochondrial dysfunction in AD is a salient biomarker that not only indicates the presence of disease before the onset of symptoms [143], but also appears to be the coveted goal of pharmacological strategies aimed at modulating this process [132]. Hence, potential agents and drugs with ETC/OXPHOS targeting capabilities may represent a strategy against AD.

In a very recent and interesting review study [144], data are collected regarding mitochondrial complexes of ETC involved in OXPHOS which can function as a hub for small molecule-targeted therapeutics that provide improved mitochondrial activity and increased cellular energy are collected. Notably, the partial inhibition of the mitochondrial complex I (CI, NADH: ubiquinone oxidoreductase) with small molecules, i.e., metformin (MET), resveratrol (RSV), berberine and epigallocatechin-3-gallate (EGCG), has emerged as a therapeutic strategy for multiple human conditions, such as cancer and neurodegenerative diseases, including AD [144]. In detail, all four CI inhibitors have been observed to penetrate the BBB, although their pharmacological properties and bioavailability need to be improved in order to increase therapeutic efficacy. In fact, the therapeutic action of RSV, berberine and EGCG turns out to be limited, possibly due to poor stability, short half-life and very low bioavailability (<1%), in contrast to MET [144].

Among the molecules that have been explored to treat AD and that target ETC is J147, a curcumin derivative that prevents or slows the development of neurodegeneration in AD [145]: its administration improves memory, restores cognition and maintains synaptic protein levels in APP/PS1 mice containing human transgenes for both APPswe and PSEN1 containing an L166P mutation. J147 also protects against age-associated brain toxicities by targeting the α-F1 subunit of ATP synthase and subsequently modulating the AMPK/mTOR (mammalian target of rapamycin) pathway [146].

Mitochondrial-targeted analogs of coenzyme Q (CoQ) are under development to promote OXPHOS and to reduce oxidative damage. In vitro and in vivo studies support the neuroprotective potential of CoQ10 in AD: it reduces Aβ plaque deposition and alleviates memory deficits in AD transgenic mice [147]. Unfortunately, clinical evidence currently does not support the efficacy of CoQ10 or its derivatives to treat AD [122].

Authors examined the bioenergetic effects of two mitochondrial-targeted CoQ analogs in their quinol forms, mitoquinol (MitoQ) and plastoquinonyl-decyl-triphenylphosphonium (SkQ1). MitoQ, which localizes in mitochondria, mitigates AD symptoms, including synaptic loss, Aβ accumulation and OS in 3xTg-AD [148]. At nanomolar concentrations, SkQ1 (plastoquinonyl-decyltriphenylphosphonium) mitigates age-related decline and reduces pathological accumulation of Aβ and hyperphosphorylation of Tau in an OXYS rat model, a suitable model of sporadic AD [149].

SS-31, an opioid agonist peptide, promotes the production of ATP by binding to cardiolipin, a phospholipid necessary for the activity of proteins embedded in MMI [122]. SS-31 improves mitochondrial dysfunction and OS in primary neurons of APP-expressing mice; in vivo, 6-week intraperitoneal injection of SS-31 inhibits Aβ production and preserves mitochondrial function and synaptic activity in APP-carrying mice [150].

S-equol, an estrogen receptor β agonist derived from daidzein, promotes mitochondrial respiration and glycolytic flux in hippocampal neurons and increases COX1 activity and expression in the brains of ovariectomized mice [151].

Furthermore—in light of the fact that AMPK improves energy metabolism through upregulation of glucose uptake, glycolysis and mitochondrial biogenesis—5-aminoimidazole-4-carboxamide ribonucleotide (AICAR), a specific activator of AMPK [152], has been proven to inhibit Aβ secretion from primary AD mouse neurons and its effect is counteracted by compound C, an AMPK inhibitor [153]. These data suggest a mechanistic connection between the etiology of AD and impaired metabolic signaling and also suggest that strategies that promote the activation of pathways that regulate brain bioenergetic function and glucose metabolism may actually represent promising therapies for AD.

Even the introduction of alternative energy sources (“biofuels”) could potentially alleviate bioenergetic dysfunction in AD [154]. Acetyl-L-carnitine (ALC), an endogenous component of the MIM able to readily cross the BBB, provides acetyl groups to facilitate the synthesis of acetyl-CoA—a compound necessary for the TCA cycle and for the production of NADH and FADH_2_, which donate electrons to ETC—thus avoiding the need for PDH [154]. Furthermore, ALC increases glutathione production [154].

### 3.2. Therapeutic Strategies Targeting Mitochondrial Biogenesis

Altered mitochondrial biogenesis and reduced copy number of mtDNA are usually observed in AD [155,156]. Mitochondrial biogenesis is a primary event in maintaining the number of mitochondria and cell renewal, in adapting to cell damage and in meeting the demand for energy supply. Overexpression of PGC-1α, the gatekeeper of mitochondrial biogenesis, could reduce mitochondrial damage and improve mitochondrial biogenesis [145], as well as peroxisome proliferator-activated receptor-c (PPARc) can regulate mitochondrial energy metabolism and induce mitochondrial biogenesis [157]. As a result, intelligent drug development targeting PGC-1α and PPARc can be useful for treating AD.

Thiazolidinedione (TZD)—tested in vitro and in vivo as a PPARc agonist—improves cognition in AD patients through its ability to enhance mitochondrial function [158]. Recently, Hamano et al. [159] found that pioglitazone—an active substance belonging to the class of thiazolidinediones—reduces p-Tau levels, lowers hippocampal Aβ levels and inactivates glycogen synthase kinase 3β in a cell line that overexpresses the Tau protein. Furthermore, Sato et al. [160], conducting a 6-month, randomized, open-controlled trial to test the effect of PPAR-γ agonist pioglitazone in patients with mild AD, accompanied with type II diabetes mellitus, demonstrated that the treatment with pioglitazone exhibited cognitive and functional improvements, and stabilization of the disease in diabetic patients with AD. As an adverse side effect in this pilot study, three patients in the pioglitazone group showed mild peripheral edema, but the adverse effect was tolerated without discontinuation of the study medication.

Other examples of compounds that activate the PPAR-PGC-1α axis include rosiglitazone and bezafibrate: the former attenuates mitochondrial dysfunction by increasing mitochondrial mass, while the latter, bezafibrate, increases mitochondrial ATP production by upregulating the mitochondrial protein expression [161]. Concerning the other PPAR-γ agonist, rosiglitazone, a randomized, double-blind pilot trial demonstrated that 6 months of treatment with rosiglitazone showed improvements in memory and selective attention, associated with less reduction in plasma Aβ40 and Aβ42 of 20 patients with AD compared with 10 control patients [162]. Furthermore, SIRT1 agonists, quercetin and RSV activate sirtuins and AMPK, which stimulate the biogenesis of mitochondria [161].

An ad hoc candidate with excellent potential is RSV, widely known to act as an antioxidant agent (see below, Section 3.5). It is a polyphenol present in red grapes, increasing the activity of AMPK and PGC-1α and promoting mitochondrial biogenesis by activating SIRT1 [163]. A Phase II clinical trial (Clinicaltrials.gov, ID: NCT01504854) in patients with mild to moderate AD showed that RSV is safe and well tolerated. Therefore, the regulation of altered mitochondrial biogenesis by PGC-1α and PPARc in dysfunctional mitochondria is a propitious target for AD intervention and treatment. In APP/PS1 mice, a decline in SIRT1 expression and activity elevates senile plaques and OS, which is partly reversed by RSV treatment [122].

Another compound that leads to an improvement in mitochondrial biogenesis is TFAM: it has been designed to rapidly pass through cell membranes and center mitochondria [164]. Expression of human TFAM significantly improves cognitive function, reducing the accumulation of both 8-oxoguanine, an oxidized form of guanine, in mtDNA and intracellular Aβ in 3xTg-AD mice and increases expression of transthyretin, known to inhibit the aggregation of Aβ (for refs, see [165]). Caloric restriction also appears to be able to induce mitochondrial biogenesis, improving the lifespan of animals and preventing age-related diseases, including cognitive decline (for refs, see [165]).

### 3.3. Therapeutic Strategies Targeting Mitochondrial Dynamics

The mitochondria are constantly in motion both structurally and functionally [122], obscuring the image of the static organelle. It is precisely because of their plastic and dynamic nature and their close relationship with OS that the lack of removal of damaged mitochondria causes an increase in OS and an accumulation of dysfunctional neurons in the brains of AD patients [122]. On one side, mitochondrial fusion promotes the exchange of mtDNA, proteins, respiratory complexes and nucleoids between these organelles in order to maintain their functional integrity and fission; on the other side, it is essential for the removal of dysfunctional mitochondria after their cleavage into child ones, which are predisposed to be swallowed by autophagosomes for lysosomal degradation through mitophagy [122].

In diseases such as AD, mitochondrial fragmentation is detrimental to cellular bioenergetics. Inhibitor/s selectively targeting Drp1 could display potential therapeutic effects in AD since this fission protein has been found abnormally expressed and deregulated in different AD models increasing mitochondrial fragmentation [97]; consequently, it is not surprising that reversal of fragmentation, by inhibiting Drp1, could restore mitochondrial function.

Mitochondrial fission depends on the activation of the Drp1 protein, which, translocating from cytosol to the OMM of damaged mitochondria, induces mitochondria fragmentation [166]. In multiple neurodegenerative disease models, Drp1 expression is activated and causes excessive mitochondrial fission [89]. Several studies have indicated that Aβ-induced mitochondrial fragmentation and loss of membrane potential Δψm are associated with upregulation of Drp1 [122]. Aβ-Drp1-mediated mitochondrial fragmentation, mtDNA mutations and reduction in OXPHOS have also been observed in AD brains [122]. Consistently, Drp1 inhibition has emerged as a possible therapeutic approach: its reduction improves learning and memory and protects mitochondria from fragmentation, loss of Δψm and decline in ATP in mouse models of AD [122]. Interestingly, arrested fission, leading to the formation of inter-mitochondria nanotunnels, has been observed in both animal models and patients with AD [122]. Several compounds capable of inhibiting this process have been identified and tested both in vitro and in vivo (for a complete review, see [89]). Among them, mdivi-1 inhibits excessive mitochondrial fission and promotes fusion (for refs, see [96]); dynasore has been discovered as a specific inhibitor of dynamin-1 and -2 as well as Drp1 [167], but there are few reports on its efficacy on mitochondria [168]; the molecule DDQ [diethyl (3,4-dihydroxyphenethylamino) (quinolin-4-yl) methylphosphonate)] is able to (i) reduce mitochondrial fission, (ii) enhance fusion, (iii) induce mitochondrial biogenesis [169].

Therapeutically, mdivi-1 recovers Aβ toxicity and mitochondrial dysfunction in APP/PS1 double transgenic AD mice [165], improving ATP production and neuronal viability. Treatment with mdivi-1 blocks mitochondrial fragmentation—caused by excessive mitochondrial fission and/or reduced mitochondrial fusion [165]—in pyramidal neurons from CRND8 APP transgenic mice and in hybrid (cybrid) AD cells, as shown by an increase in Δψm and high complex IV activity and ATP levels [122], thus ensuring better mitochondrial function [170]. In addition to its direct effect in reversing bioenergetic deficits, mdivi-1 has been found to regulate neuroinflammation. Mdivi-1 also partially saves mitochondrial damage due to inactivation of PINK1 [95].

A seven amino acid peptide, P110, has recently been developed; it also potently inhibits the activation of Drp1 [122] by interrupting the Drp1/Fis1 interaction on the MOM [122], in conditions in which P110 has no effect on the integrity of the mitochondrial network in basal conditions [122]. Treatment with P110 in 5xFAD mice carrying a total of five AD-linked mutations (APPswe, APP I716V (Florida), APP V717I (London), PSEN1 M164L and L286V mutations) alleviates behavioral deficits, Aβ accumulation, energy failure and OS [122]. Mitochondrial structure and function remain intact in cultured neurons, in cells expressing APPswe and in five different AD-patient-derived fibroblasts treated with P110 [171]. The advantage of P110 is that it inhibits excessive mitochondrial fission in all tissues where excessive fission occurs without affecting basal fission, i.e., the physiological one, even if used long-term in vivo [165].

Treatment with P110 and mdivi-1 has been widely successful in various preclinical models of neurodegenerative diseases such as AD—but also Parkinson’s disease and Huntington’s disease—and is currently being studied for further clinical investigations [89].

### 3.4. Therapeutic Strategies Targeting Mitophagy

The evidence from AD studies has shown that the increasing activity of mTOR—a protein that integrates all communications that come from nutrients and growth factors, and it is the connection point between cellular signals to control growth, metabolism and even longevity in healthy cells—could lead to mitophagy deficiency in the hippocampus and other brain areas [172]. Furthermore, the change in the level of mTOR phosphorylation is positively correlated with the presence of the p-Tau protein in the brains of AD patients [173]. Rapamycin, a selective inhibitor of mTOR, increases the levels of LC3 II/I (microtubule-associated protein light chain 3) and other autophagy-related proteins and suppresses mTOR signaling, highlighting its efficacy against AD for refs see [145]. Therefore, the mTOR inhibitor strategy certainly attracts enormous interest as an approach for the therapeutic treatment of AD. 

Latrepirdine (Dimebon), an antihistamine drug, has also shown therapeutic efficacy against AD both in vitro and in vivo (for ref, see [145]), by reducing mitochondrial swelling and stabilizing Δψm (for ref, see [145]). In 2018, Eckert et al. [174] have reported that the interaction between latrepirdine and glutamate receptors in HEK cell line overexpressing mutant APPswe blocks voltage-gated calcium channels and inhibits mitochondrial permeability, thereby suppressing unnecessary mitophagy or apoptosis. Dimebon drastically inhibited the levels of mitochondrial permeability transition pore (mPTP) components, causing greater vulnerability to stressors. Therefore, inhibition of mPTP in mitophagy is considered a good therapeutic AD target for the development of novel drugs. In a phase II clinical trial enrolling patients with moderate AD, latrepirdine (compared with placebo) significantly improved cognitive function (for ref, see [145]). However, a Phase III study in the treatment of mild to moderate AD patients with Dimebon was stopped, as no significant differences between the Dimebon and placebo groups were observed (Clinicaltrials.gov, ID: NCT00912288) (for ref, see [145]). 

Although SIRT1′s role in mitochondrial biogenesis is proved, a connection between SIRT1 and mitophagy dates back to 2009, when Hwang’s group found that nicotinamide treatment of primary human fibroblasts is able to extend their replicative lifespan, apparently by accelerating the rate of mitophagic flux [175], thus improving neuron survival and neurite growth. SIRT1 modulation also supports cognitive function and neurogenesis in neurodegenerative diseases (for ref, see [145]). Therefore, the design of agents or approaches to activate SIRT1 could be useful for providing protection against AD.

A few years ago, Zhang et al. [176] reported that photobiomodulation therapy (PBMT) activates the cAMP/protein kinase A (PKA) signaling pathway (enhancing the deacetylase activity of SIRT1 against AD) in APP/PS1 neurons. PBMT-activated SIRT1 overexpression reduces Aβ production and is currently considered as hopeful therapeutic strategy for AD. In summary, the development of drugs that target or activate SIRT1 in dysfunctional mitochondria is beneficial for the intervention and treatment of AD.

In a study with animal models, it has been shown that neuronal mitophagy can be restored thanks to a natural food metabolite derived from microflora, urolithin A (UA). UA abolishes AD-related Tau hyperphosphorylation in human neuronal cells and is capable of reversing memory impairment in transgenic Tau mice [109]. Activation of UA-induced mitophagy eliminates damaged mitochondria, reduces ROS production, reduces insoluble Aβ42 and Aβ40 and improves cognitive impairment in APP/PS1 mice. It also inhibits the phosphorylation of several Tau AD-relevant epitopes such as T181, S202/T205, T231 and S262 in SH-SY5Y human neuroblastoma cells overexpressing the 2N4R, 1N4R or 2N3R Tau isoforms [109]. Significant improvements in pathological parameters through the induction of UA mitophagy [107] have also been recorded in various in vivo AD models. Treatment of *C. elegans* expressing Aβ1–42 and APP/PS1 mice with UA reduced Aβ pathology and cognitive decline through activation of PINK1/Parkin-dependent mitophagy. UA saves the structural and functional defects of mitochondria and increases the number of synapses [177]. Consistently, in recent studies, Kshirsagar et al. treated mutant Tau expressed in HT22 (mTau-HT22) cells [178] and HT22 transfected cells with mutant APP cDNA (mAPP-HT22) [179] with mitophagy enhancers and observed augmented cell survival, increased mRNA and protein levels of mitochondrial fusion, increased expression of synaptic and mitophagy genes, and reduced mitochondrial fragmentation in mitophagy enhancers treated cells. In addition, among all enhancers tested in AD, UA showed strongest protective effects. Moreover, Kshirsagar et al. [180] studied mitophagy enhancer UA alone and also in combination with green tea extract EGCG against human Aβ peptide-induced mitochondrial and synaptic, dendritic, inflammatory toxicities and behavioral changes in humanized homozygous amyloid beta knockin (hAbKI) mice of late-onset AD. They observed that mitochondrial dysfunction was significantly improved in both treated groups, accompanied by a reduction in fragmented mitochondria and increase in mitophagosomal formations in both groups, but the effect was stronger in the combination treatment, thus indicating that combination therapy is promising for the treatment of patients with late-onset AD.

The first clinical study performed on healthy sedentary elderly individuals has demonstrated the safety of UA (500 mg and 1000 mg for 4 weeks) and its benefit in modulating mitochondrial gene expression (NCT02655393) [181]. 

In addition to UA, MET and RSV, the regulators of PINK1/Parkin, also increase mitophagy [182,183]. MET promotes mitophagy through AMPK activation in vivo and in humans [107]. In PC12 cells treated with Aβ, RSV potentiates mitophagy, showing neuroprotective effects in vitro [184]. Long-term oral administration of RSV in APP/PS1 mice improves memory and mitochondrial functions, activates the SIRT1 and AMPK pathways and reduces Aβ burden [107]. However, this molecule has a serious clinical drawback, as it is metabolically unstable and, therefore, offers poor bioavailability [185].

NAD^+^, a cofactor for several proteins, including sirtuins (SIRT1, 3, 6 and 7), is directly involved in maintaining the balance between mitochondrial biogenesis and mitophagy through the NAD^+^ -SIRT1-PGC1α pathway. The reduction in NAD^+^ leads to a decrease in mitophagy and the accumulation of misfolded proteins in neurons [122]. Nicotinamide riboside (NR) and nicotinamide mononucleotide (NMN) are also strong inducers of mitophagy [107]. In *C. elegans* expressing Aβ1–42, NR induces mitophagy, reduces Aβ load and proteotoxic stress, increasing lifespan [107]; in the same model, NMN improves PINK1/Parkin-dependent mitophagy and reverses memory defects [109]. In mice, APP/PS1 NR reduces cortical deposits of Aβ, increases the mRNA levels of PINK1, LC3 and OXPHOS proteins and improves cognitive functions [107].

In APP/PS1 mice, actinonin (AC), a natural antibacterial compound, also restores the morphology and functions of mitochondria and improves the number of synapses by stimulating mitophagy [107].

Spermidine, a small natural organic molecule known to extend the lifespan of yeasts, flies, nematodes and mice by upregulating autophagy, also stimulates the PINK1/Parkin mitophagic pathway in human fibroblasts [107]. In a clinical study in elderly subjects, spermidine was found to improve hippocampal-dependent memory (NCT02755246) [186].

Furthermore, kaempferol (flavonoid) and rhapontigenin (stilbenoid) are natural compounds that induce mitophagy. They reduce p-Tau and Aβ levels in AD cells, worms or mouse models, restoring memory deficits in both study models [107]. 

In summary, several molecules are able to stimulate mitophagy, showing positive effects in preclinical AD models; however, it is still necessary to optimize these candidate drugs and, in particular, their bioavailability (i.e., biodegradation, crossing of the BBB, etc.) and their pharmacokinetics and interaction with their targets on the relevant site.

Of interest is the paper by Xie et al. [53], who have developed a screening workflow combining advanced artificial intelligence (AI) and classical laboratory approaches for identifying mitophagy modulators as potential drugs for AD treatment. The AI-driven virtual screening could have a much higher success rate than traditional screening methods for small drug compounds.

### 3.5. Therapeutic Strategies Targeting Mitochondria-Dependent Oxidative Damage

Cellular respiration is a double-edged sword. Although it is convenient, from the point of view of energy yield, to use oxygen, on the other hand, there is the generation of free radicals. Under normal conditions, antioxidants neutralize free radicals; however, their effectiveness diminishes during aging and in pathological conditions. In this regard, although antioxidants, both those derived from natural sources, which are often incorporated into dietary habits, and the exogenous ones that are administered, can play an important role in counteracting OS, thus delaying the onset and/or slowing down the progression of AD, extreme attention and care must be given to their concentration when administered, since high doses could interrupt the normal physiological process for which ROS play a leading role.

A variety of nutraceutical or pharmacological strategies have been suggested and tested to treat AD by reducing the burden of OS. It is certain that a proper diet, including vitamins, proteins and minerals will enrich the medicine for the treatment of AD [187]. Additionally, some antioxidants have showed significant effects in animal models but reduced efficacy in humans during clinical trials. For this reason, there is a lot of skepticism about the success of antioxidant therapy for AD cure.

At present, there are a number of agents, evaluated in preclinical and clinical studies, which have the potential to improve mitochondrial dysfunction [188].

Among the most studied vitamins, vitamin E is able to prevent Aβ-induced protein oxidation, ROS production and neurotoxicity in rat embryonic hippocampal neuronal culture [189], as well as preventing Aβ induction of OS and apoptosis in the rat hippocampus [190]. In addition, a notable increase in the levels of lipid peroxidation products has been observed in the cerebral cortex, cerebellum and hippocampus in mice fed with a vitamin-E-deficient diet [145]. However, in clinical settings, vitamin E, together with vitamin C, has shown limited beneficial effects on cognitive function or delayed progression of AD [122,145]. Therefore, these vitamins are often used in conjunction with other therapies based on their overall health benefits.

Curcumin, a polyphenolic compound extracted from *Curcuma longa* with anticancer, antidiabetic and antioxidant effects, upregulates the expression of the PGC-1α protein in the brain by improving ATP levels and restoring mitochondrial fusion [191]. Dietary supplementation of curcumin increases TFAM and PGC-1α expression and ATP levels in the brains of mice [192]. Furthermore, curcumin restores glutathione levels in brain tissue and reduces oxidized proteins in AD mouse models [192]. It is among those compounds that are capable of reducing Aβ accumulation, protecting mitochondria from Aβ toxicity, restoring mitochondrial function and attenuating cognitive impairment in animal models of AD (for refs, see [125]). Preclinical studies report that curcumin attenuates inflammation induced by high levels of Aβ peptides in neural tissue through activation of the PPARγ pathway, which mitigates the neuroinflammatory response via inhibiting the NF-κB signaling pathway [193], as well as protects against Tau-triggered neurotoxicity by inhibiting glycogen synthase kinase-3 [145].

N-acetyl-5-methoxytryptamine (melatonin), a primary metabolite of tryptophan, provides significant protection against OS in the brain and is more potent than vitamins C and E at physiological concentrations (for refs, see [165]). The main source of melatonin in nature is represented by foods of vegetable origin, including brown rice, corn and oats. Legumes such as lentils and beans are also rich in melatonin and, in the vegetables category, tomatoes and radishes have good amounts of it. Studies have suggested that chronic melatonin treatment attenuates memory impairment, synaptic dysfunction, OS, neuroinflammation and neurodegeneration in an aging mouse model [194]. Furthermore, preclinical results have revealed that melatonin could prevent Aβ-induced memory deficits by downregulating Tau hyperphosphorylation and modulating the phosphoinositide 3-kinase/protein kinase B (PI3K/AKT)-mediated signal cascade in the hippocampal region of mice [195]. The effects of long-term treatment with oral melatonin on APP/PS1 transgenic mice have showed that melatonin mitigated damaged mitochondrial structure and reduced the number of mitophagic vesicles in APP/PS1 mice [145].

Additionally, catechins, the bioactive components found in tea, most abundant in green tea (green tea catechins or GTC), namely epicatechin (EC), epicatechin gallate (ECG), epigallocatechin (EGC) and EGCG [192], have antioxidant properties and, more importantly, are permeable to the BBB, making them an advantageous therapeutic candidate for the treatment of AD [192]. In particular, EGCG interacts directly with Aβ peptides and prevents the formation of aggregates [192]. That EGCG and other flavonoids are ‘multipotent therapeutic agents’ that not only reduce toxic levels of brain Aβ, but also hold the potential to protect neuronal mitochondrial function in AD is confirmed by Dragicevic’s research that green tea EGCG can restore damaged mitochondrial membrane potential, ATP levels or ROS production, which decrease Alzheimer’s Aβ-induced mitochondrial dysfunction [196].

RSV, present in numerous plants, such as red grapes and blueberries, as well as in dark chocolate, has strong antioxidant properties [192], which consist in its ability to eliminate free radicals and metals, such as copper, aluminum and zinc ions [197]. In vitro and in vivo studies (for refs, see [197]) have showed that RSV has an antioxidant/neuroprotective effect in several AD models. Its antioxidant action is carried out in two ways: on the one hand, RSV reduces the formation of ROS by inhibiting the genes that code for pro-oxidant proteins, such as NADPH oxidase and myeloperoxidase, by inducing the expression of genes for several antioxidant enzymes such as superoxide dismutase (SOD), catalase (CAT), thioredoxin (TRX) and glutathione peroxidases (GPX) (for refs, see [197]); on the other hand, RSV decreases the activity of the enzymes involved in the development of OS by directly reducing the production of free radicals. Studies have also shown that RSV improves cognitive impairment by increasing glutathione levels [198]. It has been shown that RSV not only can decrease the generation of Aβ (for refs, see [197]), but also reduce, when it is incubated with Aβ, the length and number of fibrils and Aβ plaques (for refs, see [197]). In this context, RSV has a potent anti-amyloidogenic activity, reducing the levels of Aβ produced, and increases the level of neprilysin (NEP), the amyloid-degrading enzyme, in mouse models of AD [199]. However, although it is able to cross the BBB (for refs, see [197]), the clinical outcomes of RSV are ambiguous due to the low oral bioavailability (<1%), which limits its efficacy. Therefore, new approaches should be developed in order to improve the relationship between dose, bioavailability and efficacy in the human body, particularly in the brain [200]. This is why different systems of administration of drugs are designed to improve these intrinsic biological limits, such as nanoformulations (for refs, see [197]). RSV not only attenuates OS by eliminating free radicals, but also helps to eliminate Aβ by inhibiting its production and, thus, its aggregation, leading to a diminution in the production of pro-inflammatory factors and an increase in neuronal activity [201]. RSV also improves synaptic plasticity and mitochondrial dysfunction, changes the composition of the gut microbiota and prevents neuronal cell death. In addition, RSV improves the expression of several related proteins sub-serving the memory processes [202] by reducing OS. Finally, RSV appears to protect PC12 cells from Aβ-induced cytotoxicity, cell death and intracellular ROS accumulation [203]. It also inhibits membrane lipid peroxidation by decreasing the toxic effects produced by ROS.

Nevertheless, there are potential interventions to restore or improve mitochondrial function using antioxidant therapies aimed solely at the mitochondria. Targeting harmful ROS without affecting ROS signaling would be ideal in the clinical management of AD. These mitochondria-targeted antioxidant molecules confer greater protection against oxidative damage in mitochondria than non-targeted cellular antioxidants and have shown a hundredfold rapid entry into mitochondria compared to other natural antioxidants [204]. 

There are several antioxidant compounds on the market that selectively accumulate inside the mitochondria and scavenge free radicals. These include MitoQ (ubiquinone), MitoVitE (a derivative of mitochondrially targeted vitamin E), MitoPBN (a derivative of α-phenyl-N-tert-butyl nitrone), MitoTEMPOL (mimetic superoxide dismutase), SS-tetra peptides (SS31), MitoPeroxidase [205] and others. Melatonin itself, an ancient antioxidant (see above), can also enter the mitochondria specifically, through oligopeptide transporters, PEPT1 and PEPT2, by promptly repairing its pro-oxidant environment [205]. A favorable therapeutic antioxidant that is able to easily cross the BBB and has been successfully targeted to mitochondria is MitoQ [206]. It consists of a modified ubiquinone conjugated to a triphenylphosphonium (TPP). Conjugation to TPP allows the compound to easily penetrate the lipid bilayers and accumulate on the MIM which is negatively charged [206]. MitoQ, when localized in the mitochondria, helps to protect neurons from age-related insults by converting H_2_O_2_ into H_2_O and O_2_, thus reducing the toxic local generation of free radicals. At the same time, by mimicking the endogenous mitochondrial CoQ [207], MitoQ increases the activity of the same CoQ in the MIM. It has been observed that 5 months of treatment with MitoQ among young (2–7 months) and old (18 months) 3xTg-AD mice improves memory retention and synaptic loss, as well as decreases Aβ accumulation and Tau hyperphosphorylation [205]. In 3xTg-AD mice, treatment with MitoQ induces an improved behavioral phenotype [148]. Isolated brains from MitoQ-treated transgenic mice display reduced lipid peroxidation, Aβ load and caspase activation. MitoQ has also been reported to enhance neurite growth in in vitro and in vivo AD models [148,204].

### 3.6. The Use of Nanoparticles Targeting Mitochondria in the Treatment of AD

**Figure 2:**
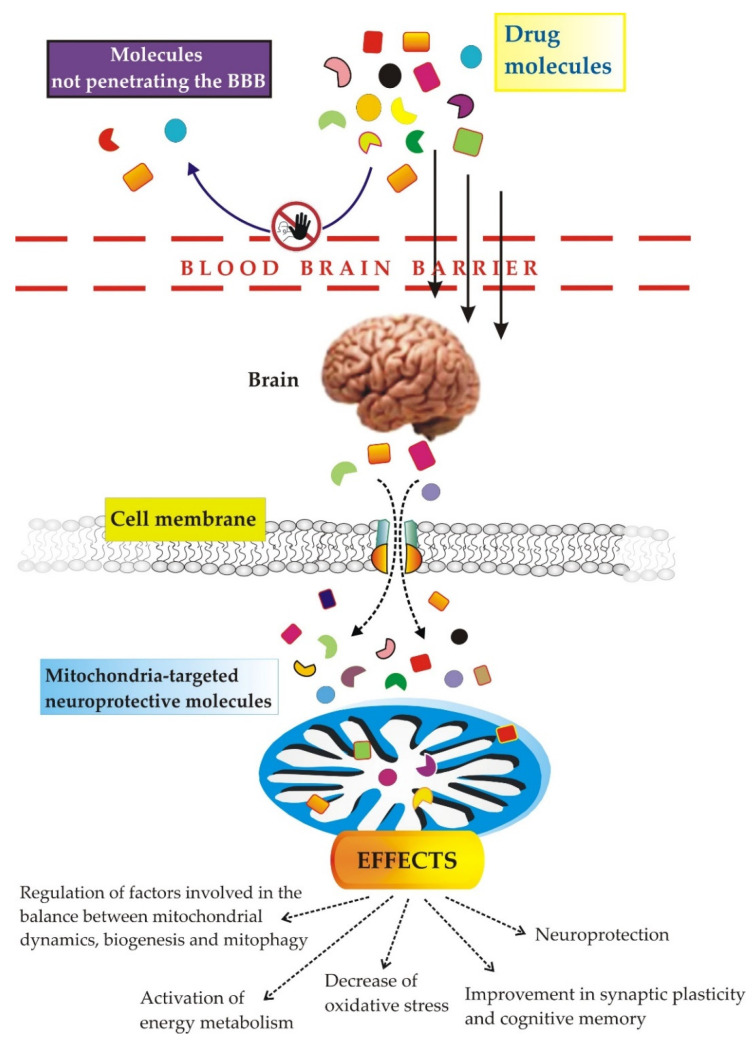
The image depicts the biological barriers, namely the BBB, the cell membrane and the mitochondrial membranes, that mitochondria-targeted neuroprotective molecules must overcome to reach neuronal mitochondria in vivo. For details, see the text.

Several obstacles, including the BBB and cell/mitochondrial membranes, reduce the efficiency of drug entry into target lesions (see Figure 2). 

The BBB works as the brain’s endogenous defense system, preventing the access to the brain of non-lipophilic and high molecular weight compounds; once a drug/compound has crossed the lipid membranes of the BBB, it will be in the aqueous environment of the brain before reaching its therapeutic goal. Therefore, the solubility of the substance must also have a ‘right degree’ of fat solubility so as not to remain trapped within the BBB [187]. It should also be considered that absorption is limited, since transport occurs via saturable transport systems [187]. The BBB also contains transporters that remove compounds from the brain, so these transport systems could reduce the effectiveness of some therapies by determining the efflux of the therapeutics themselves [187]. In AD, for example, Aβ deposition damages the BBB and reduces the Aβ efflux itself, contributing to disease progression [187]. At the same time, BBB dysregulation can be further aggravated by OS, either directly or because OS per se stimulates the deleterious effects of the Aβ peptide.

Recent treatments based on nanotechnology show particularly promising results. Nanoparticles (NPs), thanks to their adjustable size, appropriate charge and lipophilic surface, could represent an ideal system for traversing the BBB and targeting neuronal mitochondria, once administered orally, intranasally and/or parenterally (for refs, see [208]). Therefore, in this last part of the review, we will discuss physiological barriers, a challenge for treatment strategies. There are three biological barriers that drugs must overcome to reach neuronal mitochondria in vivo: the BBB, the cell membrane and the mitochondrial membrane (for refs, see [208]).

- Most drugs do not cross the BBB [208,209]: this is why various efforts have been made to overcome the BBB. Apart from parenteral administration, according to which the drug would be injected directly into the spinal fluid [208], other methods involve chemical modification of the drug, the use of viruses/exosomes as vectors for the administration of the drug to the central nervous system [210,211], intranasal administration, in order to circumvent the obstacle of the BBB [208], the destruction of the BBB by ultrasound or radiotherapy [212] and finally, strategies based on nanotechnology.
- The other barrier to drug administration inside the cell is the cell membrane, which is a lipophilic membrane, therefore, lipophilic and small molecular weight drugs can cross it [213]. Otherwise, other drugs can enter cells through the macropinocytosis, caveolae and clathrin pathways [214].
- Selectively targeting drugs to mitochondria in vivo [208] needs to take into account the existence of a proton gradient (negative inner Δψm), which produces a strong negative potential on the IMM of approximately −160–180 mV [208] and influences the entry of the drug into the mitochondria, the presence of phospholipid cardiolipin and, last but not least, the impermeability of the mitochondria. If we add to all this the fact that four parts are distinguishable in the mitochondria, namely the MOM, the MIM, the intermembrane space (IMS) and the mitochondrial matrix, the task becomes very difficult. Both high and small molecular weight lipophilic particles (< and >500 mw) can freely penetrate through the MOM, but the Δψm makes the MIM resistant to anionic molecules [215]. Therefore, these barriers prevent the potential drug from being delivered to the mitochondrial space.

Nanotechnology-based targeted delivery to mitochondria may be an effective approach to treat mitochondrial dysfunctions in AD. NPs must be carefully designed when they are intended for a dual targeting system, simultaneously targeting one of neurons and mitochondria.

There are several types of NPs designed to encapsulate therapeutic drugs based on the characteristics of the barriers: (*i*) liposomes and also polymeric NPs are synthesized because mitochondrial membranes are rich in cardiolipin; (*ii*) cationic molecules, such as dequalinium (DQA) and TPP, are synthesized on the basis of the strong negative membrane potential of the IMM; (*iii*) BBB-penetrating peptides, such as neural cell adhesion molecule mimetic peptide C3 and a short peptide derived from rabies virus glycoprotein (RVG), RVG-29, are modified on the NPs surface to improve BBB overcoming and neuronal targeting capacity [208]. Furthermore, the physical properties of the NPs, i.e., the size, shape and surface charge, certainly affect the targeted delivery. Neurotoxicity and neural uptake of NPs also need to be further explored in order to preserve both normal brain regions and the function of other organs by spreading through the circulatory system. The mitochondrial targeting molecules themselves should be designed to improve their efficiency, i.e., ensure drug accumulation and physiological outcomes on the mitochondria. The route of excretion and the time of systemic circulation must be added to all this work program of the NPs. Last but not least, what is needed to verify the effects of NPs in humans is clinical study.

### 3.7. Intercellular Mitochondrial Transfer as Potential Therapeutic Approach for AD

More and more research suggests that mitochondria communicate and collaborate with each other, both within the same cell and between different cells, influencing the health of the organism. To further stress the great potentialities of mitochondria-based therapies, it is worth reminding that healthy mitochondria from donor cells, through particular mechanisms (for refs, see [216]), are able to fully restore metabolic and energetic function when transplanted into recipient cells containing damaged ones [217].

Surprisingly, Nitzan and coworkers, using active and intact mitochondria, observed that functionally active mitochondria transfer, i.e., mitochondria acting as whole organelles rather than just targeting one of their abnormal functions, is beneficial for treating cognitive deficits, brain pathology and mitochondrial defects in a mouse model of AD [218]. In detail, following 2 weeks of intravenous injection with fresh isolated human mitochondria, AD mice show significantly improved cognitive performance almost comparable to that of untreated control mice in the absence of any obvious toxicity [218].

## 4. Conclusions

So far, none of the treatments for AD have been validated to cure or reduce the progression of the disease. Thus, it is urgent and indispensable to find new therapeutic strategies. In this context, while considering the effort employed by many in the attempt to identify the winning therapeutic target, we believe it is important to highlight that these studies have several limitations, including a relatively small number of participants enrolled, usage of different experimental techniques, as well as analysis of different types of samples, such as peripheral blood cells from living AD patients or *post mortem* brains, rather than animal models. The lack of specific models is certainly the biggest obstacle that AD research has always encountered. It is also important to consider that the changes observed in post mortem studies may be a consequence of the evolution of the disease and may not be present at the beginning of the disease itself; therefore, these changes are potentially unsuitable to be candidates as biomarkers or, at the same time, therapeutic targets.

Since dementia affects a large number of people, acting on individual risk factors could represent effective prevention on a global level. It involves acting on elements that can be controlled in whole or in part, while age and genetics are by definition not modifiable. Instead, there are some conditions, in particular those related to lifestyle, on which it is possible to intervene, obtaining significant results on a clinical level. Since they are habits that exert their negative effect slowly over time, interventions are also measurable in the long term and only accurate epidemiological studies allow us to highlight both risks and benefits, such as, for example, a low level of education, the carrying out of manual work activities, a scarcity of social relationships [219,220]. Longitudinal studies have highlighted their ability to increase the prevalence of dementia in the population. On the contrary, there is no intervention study that allows to start a prevention plan, also because it is a question of vital conditions that are often irremediable, inherent in human life. A richer society in terms of social relations, culture and also with respect to the economic potential of citizens could guarantee a brighter future for all. However, this is not a preventive plan, but a way to improve the conditions of coexistence for all.

One more reflection: new therapies have long development times; therefore, in the short and medium term it is not possible to replace the drugs approved today with molecules capable of modifying the course of the disease. Therefore, it is necessary to make the most of what is available, trying to achieve synergies where possible (including between pharmacological therapies and non-pharmacological approaches), requiring indispensable preliminary checks validating the use of multiple coordinated interventions as more effective than monotherapy. Recently, for example, researchers have begun to explore the use of antioxidants in combination with other antioxidant compounds, as well as drugs currently used to treat neurodegenerative diseases [187]. This is referred to a combination or conjugate therapy. However, it cannot be excluded that adverse effects may occur when more than one compound is administered, as the substances can affect each other. Complex formation or competition for transport systems in the cell may occur, especially when moving from an in vitro to an in vivo experiment and even more when the hypothetical combined therapy passes from the laboratory to clinical trials on patients.

As things currently stand, targeting aspects of mitochondrial dysfunction (see Section 2) may present viable therapeutic approaches to combat the signs of AD. The function of mitochondria goes far beyond the already known role of the cell’s powerhouse: all mitochondria are surprisingly interdependent and, in different tissues, take on several other specialized tasks. Therefore, an understanding of the vibrant signaling networks formed by mitochondria in and out of neurons could help unlock various secrets about this progressive complex, multifactorial, neurodegenerative disease that imposes a growing burden on healthcare systems.

## Data Availability

Not applicable.
